# Significant Increase in Oxidative Stress Indices in Erythrocyte Membranes of Obese Patients with Metabolically-Associated Fatty Liver Disease

**DOI:** 10.3390/jpm14030315

**Published:** 2024-03-18

**Authors:** Valeria Tutino, Valentina De Nunzio, Rossella Donghia, Emanuela Aloisio Caruso, Anna Maria Cisternino, Palma Aurelia Iacovazzi, Anna Margherita Mastrosimini, Elizabeth Alicia Fernandez, Vito Giannuzzi, Maria Notarnicola

**Affiliations:** 1 Laboratory of Clinical Pathology, National Institute of Gastroenterology IRCCS “Saverio de Bellis”, 70013 Castellana Grotte, Italyanna.mastrosimini@irccsdebellis.it (A.M.M.); 2 Laboratory of Nutritional Biochemistry, National Institute of Gastroenterology IRCCS “Saverio de Bellis”, 70013 Castellana Grotte, Italy; 3 Data Science Unit, National Institute of Gastroenterology IRCCS “Saverio de Bellis”, 70013 Castellana Grotte, Italy; rossella.donghia@irccsdebellis.it; 4 Ambulatory of Clinical Nutrition, National Institute of Gastroenterology IRCCS “Saverio de Bellis”, 70013 Castellana Grotte, Italy; 5 Unit of Technology Transfer, National Institute of Gastroenterology IRCCS “Saverio de Bellis”, 70013 Castellana Grotte, Italy; 6 Department of Gastroenterology, National Institute of Gastroenterology IRCCS “Saverio de Bellis”, 70013 Castellana Grotte, Italy

**Keywords:** MAFLD, peroxidation index, fatty acid profile, erythrocyte membranes

## Abstract

Metabolic dysfunction-associated hepatic steatosis (MAFLD) indicates the metabolic risk associated with hepatic steatosis, overweight and obesity, and clinical evidence of metabolic dysregulation. Since MAFLD is one of the diseases that show a high frequency of alterations in the lipid content of cell membranes, the aim of this study was to evaluate the indices of oxidative damage of erythrocyte membranes in overweight and obese MAFLD subjects. The study was conducted on serum samples and red blood cell membranes of overweight and obese MAFLD subjects. For each patient, biochemical measurements and lipidomic analyses of erythrocytes membranes were performed. Significant differences in fatty acid profiles of RBC membranes were found between overweight and obese patients. In particular, the Peroxidation Index (PI) was higher in the erythrocyte membranes of obese subjects than in overweight subjects. The same behavior was observed for Unsaturation Index (UI) and Free Radical Stress Index (Free RSI), supporting the fact that the systemic increase in oxidative stress was associated with obesity. The study shows that there is a different susceptibility to erythrocyte membrane peroxidation for overweight and obese subjects, and the increased values of oxidative stress indices observed in the erythrocyte membranes of obese patients with MAFLD may be a possible indicator of pro-oxidative events occurring in obesity-related diseases.

## 1. Introduction

Metabolic dysfunction-associated fatty liver disease (MAFLD) emphasizes the metabolic risk associated with liver steatosis, characterized by a pathologic accumulation of fat inside the hepatocytes (mainly as triglycerides), strongly also linked to insulin resistance, obesity and overweight [1]. No effective pharmacological intervention against MAFLD is currently adopted, so diet and lifestyle modifications for weight control are preferably used to counteract the metabolic alterations that occur in MAFLD [1,2]. The identification of the mechanisms involved in the progression of this disease is important to reveal novel biomarkers and therapeutic targets.

Although many factors contribute to the pathophysiology of MAFLD, several studies show that lipotoxicity is a key factor, together with chronic inflammatory tissue conditions [3]. Lipid peroxidation produced by oxidative stress activates inflammatory pathways contributing to an upregulation of reactive oxygen species (ROS) [4]. 

Literature data demonstrate that in pathological conditions, lipid peroxidation is a mechanism through which cells respond to ROS [5]. The most susceptible to peroxidation are the fatty acids with a high number of double bonds, such as the polyunsaturated fatty acids (PUFAs) located in cell membranes [6]. 

The erythrocyte membrane might be a valid biomarker for the fatty acid composition present in other body cells. Several studies have shown that changes in the fatty acid composition of red blood cell (RBC) membranes have been observed in different metabolic diseases, including Nonalcoholic Fatty Liver Disease (NAFLD) and colorectal cancer (CRC) [7,8,9,10]. In this regard, we have previously found low levels of Saturation Index, given by the relationship of stearic acid to oleic acid in RBC membranes of patients with severe Nonalcoholic Fatty Liver Disease (NAFLD) compared to controls [11]. In addition, high levels of arachidonic acid (AA) were observed in erythrocyte membranes of NAFLD patients, where the AA/EPA ratio levels (arachidonic acid/eicosapaentenoic acid) were associated with liver injury [7]. AA is a polyunsaturated fatty acid belonging to the omega-6 family and constitutes the main precursor of eicosanoids, substances involved in the inflammatory response [7].

The fatty acid profile of red blood cells has been tested in various studies, in the context of NAFLD [8,12]. Interestingly, molecular alterations in erythrocyte membranes seem be mediators of disease [12] and, recently, experimental studies have proved that red blood cells are active players during immunometabolic dysregulation [13]. 

There is also evidence that a fatty acid dysregulation in erythrocyte membranes is associated with fibrosis-related liver disease, supporting the central role of erythrocytes in the onset of different metabolic alterations [14]. Alterations in the biophysical properties of the red blood cell membrane contribute to modifying the fluidity and permeability of the membrane itself, causing damage to the cells.

Lipid peroxidation into the cell membranes can have a significant impact on the intensity of oxidative stress and inflammation inside the cells [6,15]. The increase in inflammatory molecules, associated with a down-regulation of cellular antioxidant system, seems to induce a dysregulation of the lipid metabolism. Altered expression levels of fibroblast growth factor 21 (FGF21) have been detected in patients with metabolic syndrome [16]. FGF21 is a hormone involved in the regulation of lipid and glucose metabolism and its expression is often associated with obesity and liver steatosis [17]. 

Moreover, an overexpression of modified lipoproteins, particularly oxidized- and glycated- low-density lipoproteins (LDL) has been detected in NAFLD subjects [18]. The increased prevalence of oxidized LDL (oxLDL) in the serum is often related to higher body mass index (BMI) [19], and likely to an inflammatory state, consistent with an increased metabolic risk for patients with NAFLD. 

Several studies have demonstrated that both the quantity of LDL and the quality (particularly small, dense LDL) may increase the metabolic risk [20,21].

Recently, we observed high levels of small dense LDL (sdLDL) particles in the serum of CRC patients, and this altered pattern was also associated with higher serum levels of oxLDL [22].

sdLDL has reduced binding capacities to LDL receptors showing a stronger affinity to extracellular matrix and a major tendency to oxidative modification [23]. sdLDL particles also play a central role in both the initiation and progression of tissue inflammation through platelet activation and vascular endothelial cell injury [24]. In particular, the smaller LDL fractions show greater trans endothelial transport and an increased oxidative susceptibility [25].

Since MAFLD is one of the pathologies that show a high frequency of the alterations in the lipid content of cell membranes, the aim of this study was to evaluate the fatty acid profile, particularly the indices of oxidative stress in the erythrocyte membranes from overweight and obese MAFLD subjects. The study was performed to identify biochemical parameters capable of discriminating a subset of patients who could benefit from nutritional or dietary interventions.

## 2. Materials and Methods

### 2.1. Patients

The study was conducted on 42 subjects with a sedentary lifestyle (26 male and 16 female), aged between 30 and 60, recruited on a voluntary basis by the Clinical Nutrition Clinic of our Institute. The inclusion criterion was the diagnosis of metabolically associated fatty liver disease (MAFLD) based on clinical, instrumental, and serum parameters. Specifically, all subjects had a controlled attenuation parameter (CAP) ≥ 300 dB/m, measured by vibration-controlled elastography (VCTE) implemented on FibroScan^®^ (Echosens, Paris, France) and BMI ≥ 25 kg/m^2^, calculated as weight (kg) divided by the squared height (m^2^). The BMI was taken as a reference to define the two categories: overweight patients had 25 ≤ BMI ≥ 29.99 and obese patients had BMI ≥ 30. Exclusion criteria were the following: gastroesophageal reflux disease; inflammatory bowel disease; oncological diseases; serious medical conditions that could compromise participation in the study; people on a special diet or using blood thinners; and subjects unable to follow a diet for religious or other reasons. Written informed consent was obtained from all subjects for blood tests and clinical data collection.

### 2.2. Eating Habits

To evaluate eating habits, all participants were interviewed by nutritionists. During the personal interview, subjects reported the foods they usually ate, the weekly frequency and the quantity. To facilitate recording the amount of food consumed, participants were presented with pictures of 3 different portions of each food they cooked. For other foods, however, quantities were recorded in household units or volume. Special attention was paid to the preparation of the dishes, the type of food used, cooking practices, the use of spices, and the amount and type of oil used. Food consumption was converted to energy using metaDIETA software, version PROFESSIONAL 4.0.1 (METEDA srl, Rome (RM), Italy).

In addition, metaDIETA made it possible to calculate the percentage and/or grams of macro- and micronutrients routinely consumed by patients. 

### 2.3. Biochemical Measurements

Blood samples were collected from all participants after a 12 h fast in tubes containing ethylenediaminetetraacetic acid anticoagulant (EDTA) for blood count analysis or silica gel as clotting activator for serum separation. Serum samples were separated by centrifugation at 3200 rpm for 10 min, and used appropriately.

Biochemical measurements were performed at the Laboratory of Clinical Pathology of our Institute. Complete blood count, glucose, insulin, HOMA test, total cholesterol, high density lipoproteins (HDL), low-density lipoproteins (LDL), triglycerides, aspartate aminotransferase (AST), alanine aminotransferase (ALT), gamma-glutamyltransferase (GGT), alkaline phosphatase (ALP), protein C-reactive (PCR), and ferritin were assayed by sets of XN-1000 (Sysmex, Norderstedt, Germany) and Cobas 8000 (Roche diagnostics S.p.A., Monza, Italy) autoanalyzers, respectively.

### 2.4. Red Blood Cell Membrane Fatty Acid Profile

The blood sample in the tube with EDTA was used for red blood cell (RBC) membrane lipidomic analysis. Briefly, 500 μL of whole blood was centrifuged at 4000 rpm for 5 min at 4 °C. The plasma was removed and mature cells, having a smaller diameter and higher weight, were isolated using an automated protocol (Robot LNG-R1, Lipinutragen-Tecan, Bologna, Italy). Subsequently, cell lysis, isolation of the membrane pellet, extraction of phospholipids performed according to the method of Bligh and Dryer [26], and transesterification of FAMEs with a solution of potassium hydroxide (KOH)/methyl alcohol (MeOH) (0.5 mol/L) were performed with the Robot LNG-R1. After extraction of the FAMEs with hexane, the esterified FAMEs were analyzed with a gas chromatograph on a system equipped with a splitless inlet, an FID detector, and a hydrogen gas generator (Thermo Fisher Scientific, Milan, Italy). A total of 1 μL of FAME was carried out on BPX70 0.25 UM capillary column SGE analytical science (SGE EUROPE Ltd., Kiln Farm Milton Keynes, UK). Hydrogen was used as carrier gas (3.0 mL min^−1^, constant flow mode). The temperature of the injector and the FID detector was 250 °C.

Fatty acid (FA) quantification was calculated as relative % (each FA as total FA content). Peaks were identified by comparing them with a mixture of standards (Supelco 37-Component FAME Mix, Sigma Aldrich, Milan, Italy). A representative chromatogram with the retention times of single detected fatty acids is shown in Figure S1.

Of the 37 FAs present in the RBC membranes analyzed, we paid attention to a few components representative of the three main FA families: saturated fatty acids (SFAs), especially palmitic and stearic acid; monounsaturated fatty acids (MUFAs), such as palmitoleic, oleic, and cis-vaccenic acid; polyunsaturated fatty acids (PUFAs), in particular linoleic (LA), arachidonic (AA) and dihomo-gamma-linolenic (DGLA) acid belonging to the omega-6 PUFAs family; and eicosapentaenoic (EPA), docosapentaenoic (DPA) and docosahexaenoic (DHA) acid, belonging to the omega-3 PUFAs family.

Taking into account the mentioned FAs, some indices were calculated: the Peroxidation Index (PI) [(% MUFA × 0.025) + (% LA × 1) + (% DGLA × 2) + (% AA × 4) + (% EPA × 6) + (% DHA × 8)], the Unsaturation Index (UI) [(% MUFA × 1) + (% LA × 2) + (% DGLA × 3) + (% AA × 4) + (% EPA × 5) + (% DHA × 6)], the Free Radical Stress Index (Free RSI) (oleic acid + AA) and the Saturation Index (SI) (stearic acid/oleic acid) [27].

### 2.5. Fibroblast Growth Factor 21 (FGF21) and Oxidized Low-Density Lipoprotein (oxLDL) Assay

Serum levels of fibroblast growth factor 21 (FGF21) and oxidized low-density lipoprotein (oxLDL) were measured with a quantitative sandwich ELISA kit (MyBioSource Inc., San Diego, CA, USA) according to the manufacturer’s recommendations. Briefly, the standards and serum samples were pipetted in duplicate into a 96-well plate, and the plate was incubated at 37 °C. The specific antibody was added and the plate was incubated again. After 60 min, the wells were washed, HRP conjugate reagent was added, and the plate was incubated at 37 °C. After washing, the substrate reagent was applied. The optical density (OD) was read at 450 nm and the concentration of FGF21 and oxLDL was calculated using a standard curve for each molecular target.

### 2.6. Small- and Dense-LDL (sdLDL) Score Analysis

The sdLDL score was calculated as the sum of subfractions 3-7 divided by the sum of subfractions 1-7 [((Σ LDL 3-7)/(Σ LDL1-7)) × 100]. Specifically, LDL was divided into seven subfractions using the Lipoprint LDL System (Quantimetrix, Redondo Beach, CA, USA). Briefly, 25 µL of serum was mixed with 200 µL of gel-loaded Lipoprint and deposited on top of the polyacrylamide gels. The tubes were placed for 30 min under UV light at room temperature and then electrophoresis. After the electrophoretic run the tubes were inserted into a digital scanner to acquire the image and the detected lipoprotein bands were analyzed using the Lipoware software program (Version 1.62, Quantimetrix Corporation, Redondo Beach, CA, USA).

### 2.7. Statistical Analysis

The distribution of the data was checked by the Shapiro–Wilk test. Patients’ characteristics are reported as mean and standard deviation (Mean ± SD) for continuous variables, and as frequency and percentages (%) for categorical variables. To test the association between the independent groups (overweight vs obese), a chi-square or Fisher test was used for categorical variables, where necessary, and the Wilcoxon Rank Mann–Whitney was chosen for continuous variables. To test the null hypothesis of non-association, the two-tailed probability level was set at 0.05. The analyses were conducted using StataCorp.2021 software. Release 17. College Station, TX: StataCorp LLC.

## 3. Results

Table 1 shows the clinical and biochemical features of the enrolled subjects. The parameters considered have been analyzed for the total, overweight, and obese patient cohorts.

Data analysis showed that no difference was detected between the sex and age of the two groups of patients, excluding the possibility that the age factor is related to oxidative stress.

The RBC counts, hemoglobin, and hematocrit values were significantly higher in the obese patient group than in the overweight group (Table 1, *p* = 0.04, *p* = 0.007 and *p* = 0.04, respectively). Furthermore, white blood cell count (WBC) was significantly higher in obese patients, as well as the serum levels of alanine transaminase (ALT), the aspartate aminotransferase/alanine transaminase ratio (AST/ALT ratio), gamma-glutamyl transferase (GGT), low-density lipoprotein (LDL) and triglycerides.

Significant differences in the RBC membrane fatty acid profile have been detected between overweight and obese patients (Table 2). In particular, obese patients, compared to overweight patients, showed increased values of AA, the main fatty acid of the omega-6 family. This increase has a great influence on the peroxidation index (PI), which is higher in the erythrocyte membranes of obese subjects. The same behavior was observed for the Unsaturation Index (UI) and the Free Radical Stress Indexes (Free RSI), supporting the concept that an increased systemic oxidative stress associated with obesity occurs in these patients.

Moreover, obese patients with high values of the stress oxidative indices showed higher levels of RBC, hemoglobin, hematocrit, and small dense LDL (sdLDL) than overweight subjects, although these values were in the physiological range. In addition, significant differences were detected between the two patient groups for liver biomarkers, triglycerides, and cholesterol metabolism.

**Figure 1:**
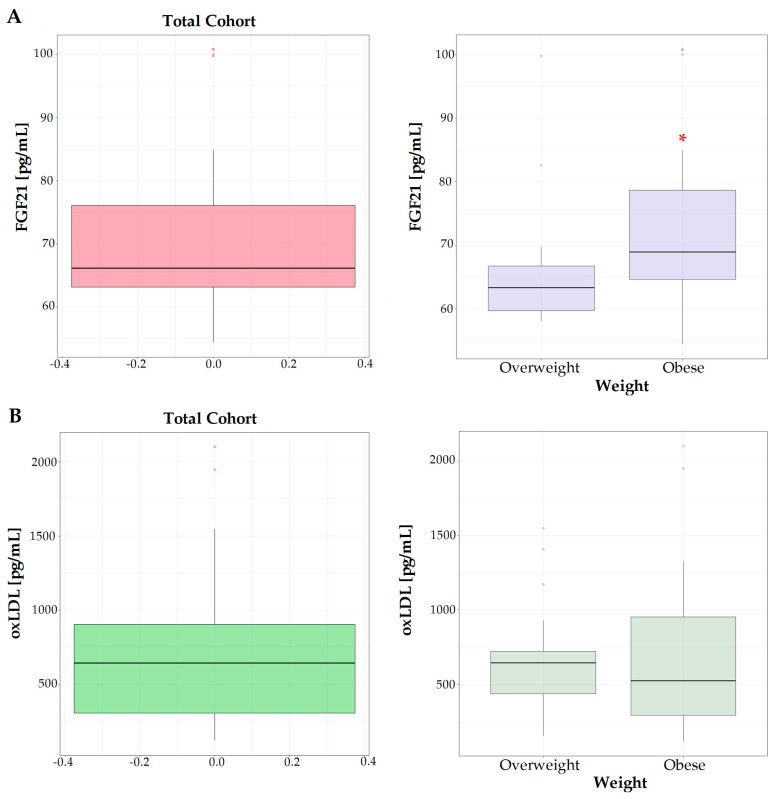
Serum levels of FGF21 (**A**) and oxLDL (**B**) in total cohort studied, overweight and obese subjects. * means a *p* < 0.05.

The levels of FGF21 were significantly higher in the serum of the obese group with respect to overweight subjects (Figure 1A), showing the involvement of FGF21 in the alterations of lipid metabolism occurring in obesity-related diseases. This growth factor is mainly exported into the circulation by the liver responding to stress or dietary factors such as high calories or protein intake.

In the serum of obese patients, a slight increase in the oxLDL levels was observed, even if the difference with respect to overweight subjects was not statistically significant (Figure 1B).

These data confirm the fact that obese patients generally present a low grade of systemic inflammation, often associated with an alteration in lipid metabolism.

The calculation of macronutrient intake (Table 3) demonstrated that the obese subjects had higher dietary protein and lipid consumption with respect to overweight subjects. In contrast, total carbohydrate intake was significantly lower in the obese patients, probably due to a lower fruit consumption. 

No evident difference was observed between the two groups of subjects for the composition of dietary micronutrients, except for the content of vitamin E (Table 3).

## 4. Discussion

Accumulating evidence suggests that changes in cell membrane fatty acid composition are related to the development of different pathologies, including metabolic diseases [28].

The evaluation of the indices of oxidative stress of the erythrocyte membrane in MAFLD patients has provided further evidence for the role of cell membrane composition in affecting the metabolism of the entire body.

Compared to overweight subjects with MAFLD, the obese patients showed higher values of PI, UI, and Free RSI in the erythrocyte membranes, suggesting a higher probability, for these subjects, of presenting tissue inflammation and altered cell metabolism.

It is interesting to note that, in the obese patients, we detected higher levels of AA, considered to be a molecular compound linked to inflammation [29] and susceptible to peroxidation [30].

In this regard, an association between AA oxidation and BMI has been recently demonstrated [31]. Changes in hepatic n-6 PUFA content, particularly in the AA levels, predispose the subject to liver steatosis by favoring lipid synthesis, over-oxidation, secretion, and fat accumulation [32].

The contribution of erythrocytes to the immunometabolic cross-talk, mainly by linking the systemic metabolism with inflammation, has been widely described [13]. The red blood cells seem be capable of producing potent immunoregulatory metabolites in response to various stimuli [33,34]. These characteristics possibly imply that erythrocytes mediate interactions between the metabolic and the immune systems [13].

Moreover, there is evidence for the pro-inflammatory role of red blood cells in the context of systemic metabolism. Benson et al. [35] showed that erythrocytes are excellent contributors to the pathogenesis of NAFLD, reporting an increase in reactive oxygen species (ROS) in red blood cells. It is evident that these erythrocyte alterations could be responsible for important molecular changes in the liver [12], enhancing the hepatic inflammation and fibrosis development.

High levels of circulating FGF21 are often linked to different dysfunctional metabolic processes, such as obesity [36]. In accordance with these studies, we found an upregulation of this hormone in the serum of the obese group, suggesting that serum FGF21 levels are indicative of a lipid dysregulation that occurs in obese patients with MAFLD. Several studies have positively correlated serum FGF21 levels with ALT levels. ALT is one of the parameters used to indicate liver failure; in fact, our data show higher levels of ALT in obese patients compared to overweight patients [37].

In these patients, probably because of a greater presence of visceral fat, we observed significant changes in the fatty acid profile of the erythrocyte membrane.

The identification of biochemical profiles, capable of evaluating a cell oxidative injury, is important to better understand the pathogenetic mechanisms involved in obesity-related diseases.

Other studies have demonstrated that altered membrane fluidity in erythrocytes could be the expression of a more generalized systemic phenomenon [38,39,40]. The interest in examining mature erythrocytes allows for the evaluation of possible lipid profile alterations connected more to metabolism than to dietary intake [40].

In this study, it is evident that the increase in the PI detected in the obese patient group confirms the role of the peroxidation of a specific type of lipids in the pathology of several human diseases, including obesity complicated with MAFLD. The proposed mechanisms of peroxidation, in turn, leads to cell membrane damage including an increase in stress-activated pathways associated with adiposity. In obese individuals, the PI could be a valid biomarker of a prolonged stress condition, which can explain disease severity, even if more clinical studies are required to understand its value and usage in clinical practice.

Diet is a major environmental factor contributing to metabolic diseases [41]. Several studies have shown that the type of diet and the Westernization of lifestyle negatively influence the cell membrane fatty acid profile [41,42]. A balanced diet involves the following distribution of macronutrients: 40–60% carbohydrates, 10–12% proteins, and 20–35% fats. The study of the eating habits of the enrolled patients highlighted an imbalance in the percentages of macronutrients, in particular a higher protein and lipid consumption. Compared to overweight patients, obese patients made excessive use of margarine, butter and oily fruits and their derivatives such as olives, walnuts, hazelnuts, peanuts, olive oil, walnut oil, sunflower oil, and foods rich in vitamin E.

The consumption of food marketing or larger food portions causes an inflammatory status and a higher production of oxidant products. The replacing of energy intake from saturated fatty acids with an equivalent energy intake from PUFAs or MUFAs could inhibit adipose inflammasome-mediated IL1-beta secretion [43].

A diet rich in natural antioxidants and bioactive compounds, such as the Mediterranean diet, has beneficial properties on metabolic syndrome. Different studies have demonstrated that one of the mechanisms of action involved in reducing the risk for the development of metabolic diseases seen in the Mediterranean diet is the ability of some its dietary compounds to inhibit the NF-kB and consequently to reduce the secretion of proinflammatory cytokines [44]. Moreover, nutrients as polyphenols reduce inflammation and oxidative stress by affecting the levels of C-reactive protein [45].

## 5. Conclusions

The identification of specific fatty acid profiles in red blood cell membranes is useful to better understand the molecular mechanisms involved in MAFLD. 

The limited number of subjects enrolled and the lack of a control group of healthy subjects could represent some limitations of the study. However, in light of the current experimental data, the study demonstrates that the oxidative indices of the erythrocyte membranes are able to discriminate between overweight and obese subjects, confirming the role for cell membrane conditions in the pathogenesis of obesity-related diseases.

## Figures and Tables

**Table 1:** Clinical and biochemical characteristics observed in total, overweight, and obese patient cohorts.

Parameters *	Total Cohort (n = 42)	Overweight (n = 19)	Obese (n = 23)	*p* ^^^
Gender (M) (%)	26 (61.90)	9 (47.37)	17 (73.91)	0.08 ^ѱ^
Age (yrs)	47.4 ± 8.46	45.5 ± 9.44	49.0 ± 7.38	0.14
BMI (Kg/m^2^)	29.2 ± 5.52	25.3 ± 2.41	32.4 ± 5.31	<0.0001
RBC (10^6^/µL)	4.93 ± 0.51	4.77 ± 0.58	5.06 ± 0.40	0.04
Hb (g/dL)	14.7 ± 1.69	13.9 ± 1.82	15.3 ± 1.29	0.007
Hematocrit (%)	43.7 ± 4.37	42.1 ± 5.09	45.0 ± 3.25	0.04
MCV (FL)	88.8 ± 3.64	88.0 ± 2.68	89.4 ± 4.23	0.09
Platelets (10^3^/µL)	247 ± 72.29	228 ± 47.37	263 ± 85.57	0.16
WBCs (10^3^/µL)	6.00 ± 1.79	5.38 ± 1.37	6.51 ± 1.95	0.04
Glucose (mg/dL)	90.5 ± 13.62	86.7 ± 12.54	93.6 ± 13.94	0.09
Insulin (µUI/mL)	13.5 ± 5.96	11.9 ± 3.80	14.8 ± 7.10	0.15
HOMA test	3.08 ± 1.67	2.53 ± 0.88	3.54 ± 2.02	0.14
AST (U/L)	20.5 ± 6.04	19.8 ± 4.06	21.1 ± 7.33	0.84
ALT (U/L)	25.9 ± 13.24	20.9 ± 11.32	30.0 ± 13.53	0.002
AST/ALT Ratio	0.91 ± 0.35	1.09 ± 0.38	0.76 ± 0.23	0.002
GGT (U/L)	30.8 ± 23.46	21.8 ± 20.34	37.9 ± 23.70	0.0005
Cholesterol (mg/dL)	202 ± 41.24	190 ± 34.18	212 ± 44.69	0.08
HDL	49.7 ± 12.16	52.5 ± 11.60	47.4 ± 12.38	0.11
LDL (mg/dL)	129 ± 38.11	115 ± 30.52	140 ± 40.48	0.03
Triglycerides (mg/dL)	126 ± 72.83	104 ± 82.19	144 ± 60.01	0.02
sdLDL score (%)	1.96 ± 3.14	1.50 ± 2.69	2.32 ± 3.47	0.39
PCR (mg/dL)	0.23 ± 0.32	0.16 ± 0.21	0.28 ± 0.38	0.14
ALP (U/L)	67.8 ± 22.69	68.0 ± 27.88	67.7 ± 21.39	0.61
Ferritin (ng/mL)	299 ± 252.77	281 ± 181.27	307 ± 285.52	0.94

* As mean and standard deviation for continuous variables and as frequency and percentage (%) for categorical. ^^^ Wilcoxon rank-sum test (Mann–Whitney); ^ѱ^ Chi-square test. Abbreviations: BMI, Body Mass Index; RBC, Red Blood Cell; Hb, Hemoglobin; MCV, Mean Corpuscular Volume; WBCs, White Blood Cells; HOMA test, Homeostasis Model Assessment test; AST, Aspartate Aminotransferase; ALT, Alanine Transaminase; GGT, Gamma-Glutamyl Transferase; HDL, High-Density Lipoprotein; LDL, Low-Density Lipoprotein; sdLDL, small dense Low-Density Lipoprotein; PCR, Protein C-Reactive; ALP, Alkaline Phosphatase.

**Table 2:** Fatty acid (FA) profile in red blood cell (RBC) membrane in total, overweight, and obese patient cohort.

RBC Membrane FA% *	Total Cohort (n = 42)	Overweight (n = 19)	Obese (n = 23)	*p* ^^^
Saturated fatty acids				
Palmitic acid	22.0 ± 1.73	22.4 ± 1.70	21.6 ± 1.69	0.16
Stearic acid	18.0 ± 1.75	17.7 ± 2.06	18.2 ± 1.45	0.35
Monounsaturated fatty acids				
Palmitoleic acid	0.16 ± 0.15	0.18 ± 0.16	0.14 ± 0.14	0.36
Oleic acid	15.5 ± 1.37	15.6 ± 1.58	15.3 ± 1.19	0.73
cis-Vaccenic acid	0.74 ± 0.14	0.74 ± 0.13	0.73 ± 0.15	0.74
Polyunsaturated fatty acids				
LA	10.4 ± 1.41	10.8 ± 1.60	10.1 ± 1.17	0.04
DGLA	1.75 ± 0.38	1.71 ± 0.45	1.79 ± 0.32	0.14
AA	14.7 ± 1.65	13.9 ± 1.35	15.4 ± 1.58	0.003
EPA	0.58 ± 0.28	0.55 ± 0.22	0.61 ± 0.32	0.83
DPA	1.85 ± 0.34	1.79 ± 0.29	1.90 ± 0.38	0.34
DHA	3.74 ± 0.74	3.78 ± 0.64	3.71 ± 0.83	0.71
Total fatty acids				
Total SFAs	41.5 ± 2.42	41.8 ± 2.13	41.3 ± 2.65	0.37
Total MUFAs	18.0 ± 1.80	18.1 ± 2.20	17.9 ± 1.43	0.85
Total PUFAs	36.4 ± 2.23	35.8 ± 2.17	36.9 ± 2.21	0.19
Total n-9 PUFAs	15.8 ± 1.45	15.9 ± 1.71	15.8 ± 1.23	0.91
Total n-6 PUFAs	30.1 ± 2.44	29.6 ± 2.41	30.6 ± 2.41	0.16
Total n-3 PUFAs	6.23 ± 1.06	6.20 ± 0.97	6.25 ± 1.14	0.99
Fatty acid index				
SI	1.17 ± 0.15	1.15 ± 0.18	1.19 ± 0.12	0.43
SFAs/MUFAs	1.15 ± 0.09	1.17 ± 0.09	1.13 ± 0.09	0.18
AA/DHA	4.09 ± 0.98	3.78 ± 0.78	4.35 ± 1.07	0.11
AA/EPA	30.6 ± 13.62	30.0 ± 13.50	31.2 ± 13.99	0.66
Omega3 index	4.33 ± 0.90	4.34 ± 0.75	4.31 ± 1.03	0.79
n-6/n-3 PUFAs	5.00 ± 1.04	4.91 ± 0.99	5.08 ± 1.10	0.68
Free RSI	30.2 ± 1.84	29.5 ± 1.92	30.7 ± 1.61	0.04
UI	128 ± 7.13	125 ± 6.21	130 ± 7.23	0.02
PI	106 ± 8.18	103 ± 5.91	109 ± 9.08	0.05

* As mean and standard deviation for continuous variables and as frequency and percentage (%) for categorical. ^^^ Wilcoxon rank-sum test (Mann–Whitney); Abbreviations: LA, Linoleic acid; DGLA, Dihomo-gamma-linolenic acid; AA, Arachidonic acid; EPA, Eicosapentaenoic acid; DPA, Docosapentaenoic acid; DHA, Docosahexaenoic acid; SFAs, Saturated fatty acids; MUFAs, Monounsaturated fatty acids; PUFAs, Polyunsaturated fatty acids; SI, Saturation Index; Free RSI, Free Radical Stress Index; UI, Unsaturation Index; PI, Peroxidation Index.

**Table 3:** Daily intake of macro- and micro-nutrients in overweight and obese patients.

Parameters *	Total Cohort (n = 42)	Overweight (n = 19)	Obese (n = 23)	*p* ^^^
Macronutrients				
Energy intake (Kcal/day)	2057 ± 308.04	1871 ± 248.34	2210 ± 268.59	<0.0001
Proteins (g)	99.8 ± 23.12	88.4 ± 16.20	109 ± 24.02	0.005
Proteins (%)	19.4 ± 3.21	19.0 ± 2.90	20.0 ± 3.47	0.35
Lipids (g)	95.7 ± 22.21	79.4 ± 14.56	109 ± 18.04	<0.0001
Lipids (%)	41.6 ± 5.40	38.3 ± 5.00	44.4 ± 3.98	0.0001
Carbohydrates (g)	210 ± 39.56	212 ± 42.76	208 ± 37.62	0.99
Carbohydrates (%)	38.6 ± 6.35	42.4 ± 5.36	35.5 ± 5.39	0.0003
Starch (g)	84.3 ± 24.35	83.3 ± 25.44	85.1 ± 23.95	0.50
Sugars (g)	52.2 ± 21.34	59.9 ± 25.99	45.8 ± 14.21	0.04
Total Fiber (g)	10.4 ± 2.55	10.6 ± 2.50	10.3 ± 2.64	0.67
Micronutrients				
Sodium (mg)	886 ± 828.15	654 ± 149.61	1078 ± 1083.90	0.09
Potassium (mg)	1168 ± 620.39	1055 ± 331.16	1262 ± 779.33	0.65
Iron (mg)	7.16 ± 4.54	5.79 ± 1.50	8.29 ± 5.79	0.16
Calcium (mg)	443 ± 129.51	461 ± 107.77	429 ± 145.84	0.44
Phosphorus (mg)	837 ± 218.69	730 ± 179.49	924 ± 322.03	0.09
Thiamine (mg)	0.53 ± 0.35	0.45 ± 0.14	0.60 ± 0.44	0.42
Riboflavina (mg)	0.63 ± 0.20	0.60 ± 0.20	0.66 ± 0.20	0.33
Niacin (mg)	10.4 ± 5.03	9.04 ± 2.59	11.6 ± 6.22	0.25
Vitamin A (mcg)	389 ± 160.24	358 ± 126.70	415 ± 182.28	0.45
Vitamin C (mg)	44.7 ± 50.16	55.6 ± 64.35	35.7 ± 33.38	0.53
Vitamin E (mg)	8.36 ± 3.73	6.90 ± 3.63	9.57 ± 3.43	0.01

* As mean and standard deviation for continuous variables. ^^^ Wilcoxon rank-sum test (Mann–Whitney).

## Data Availability

Data are available from the corresponding author upon reasonable request.

## Supplementary Materials

The following supporting information can be downloaded at: https://www.mdpi.com/article/10.3390/jpm14030315/s1. Figure S1: Panel A shows table with the retention times of Fatty Acids; panel B shows a representative chromatogram.
