# Supplementary figures and images for: Significant Increase in Oxidative Stress Indices in Erythrocyte Membranes of Obese Patients with Metabolically-Associated Fatty Liver Disease
Source: J Pers Med. 2024 Mar 18;14(3):315. doi: 10.3390/jpm14030315 (PMC10970721; doi:10.3390/jpm14030315)

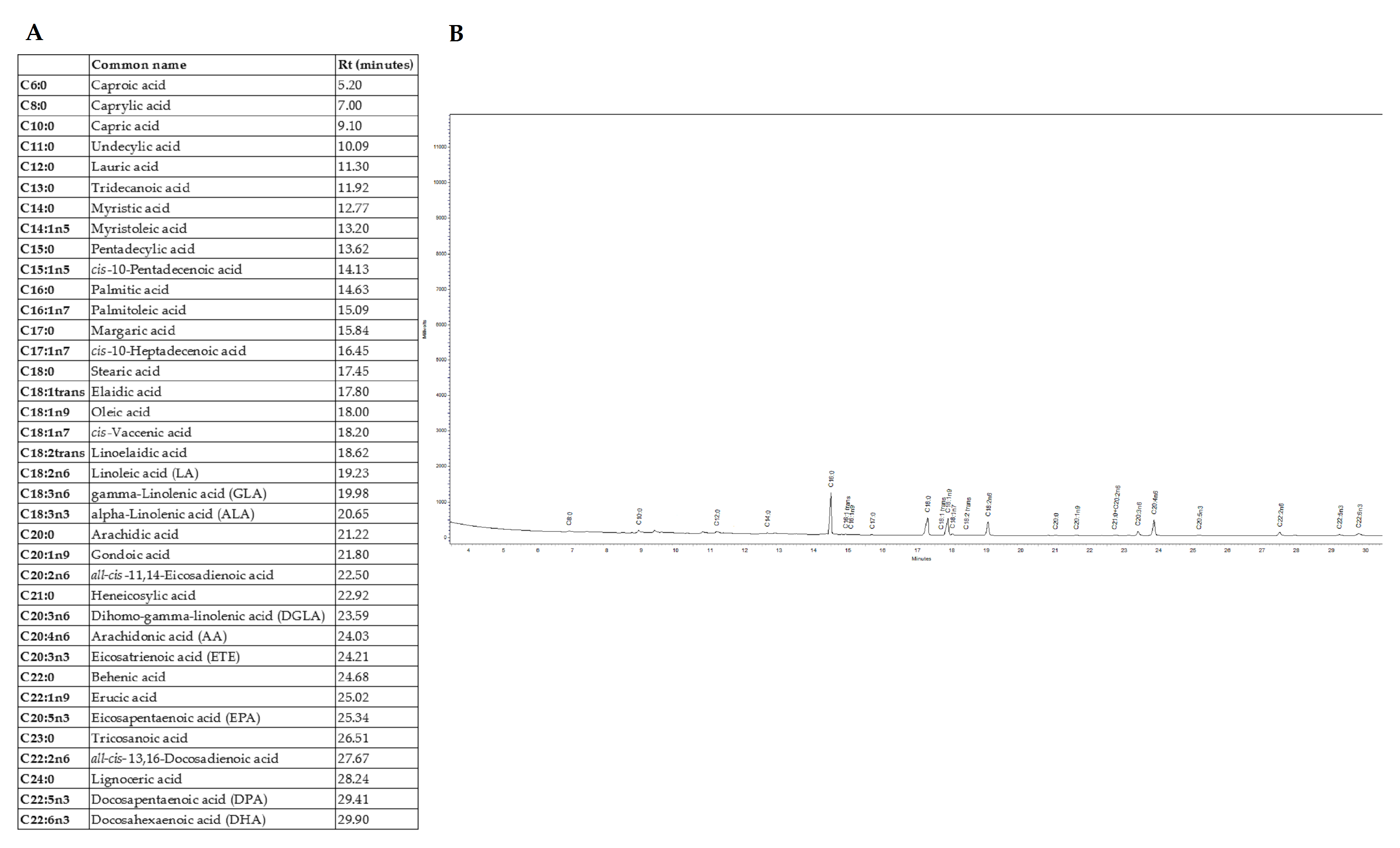

Supplement: Supplementary file 1 [file jpm-14-00315-s001.zip › Figure S1.tif]
